# A systematic review of the clinical practice guidelines for the assessment, management and treatment of eating disorders during the perinatal period

**DOI:** 10.1186/s12884-024-06995-x

**Published:** 2025-01-28

**Authors:** Chantelle Ecob, Debbie M. Smith, Zoe Tsivos, Noora Hossain, Sarah Peters

**Affiliations:** 1 Division of Psychology and Mental Health, School of Health Sciences, Faculty of Biology, Medicine and Health, The University of Manchester, 2Nd Floor Zochonis Building, Brunswick Street, Manchester, M13 9PL UK; 2 Manchester Mental Health NHS Foundation Trust, Manchester, UK; 3 Manchester Academic Health Science Centre, Manchester, UK

**Keywords:** Eating disorders, Perinatal, Pregnancy, Postpartum, Clinical practice guidelines, Guidance, Recommendations, Assessment, Management, Treatment

## Abstract

**Background:**

Eating disorders during the perinatal period can pose significant risks to both the mother and the baby. Clinical practice guidelines include statements of expected practice intending to improve effectiveness and quality of care within health care services. This systematic review aimed to identify and synthesise current clinical practice guideline recommendations on the assessment, management and treatment of eating disorders during the perinatal period.

**Methods:**

Three bibliographic databases and five guideline repository databases were searched alongside the grey literature. Guidelines were screened against eligibility criteria and recommendations for the assessment, management or treatment of eating disorders during the perinatal period were extracted. All included guidelines were assessed for quality using the AGREE-II tool. Recommendations were analysed and summarised using narrative synthesis.

**Results:**

From the 242 records screened, 17 met inclusion criteria. Guideline quality ranged from three out of seven to seven out of seven. Six overall recommendations were formed from the narrative synthesis of data: *1) Early detection: recognising the signs and symptoms, 2) Assessment and screening: a three-pronged approach, 3) Educating and supporting the mother: the importance of knowledge, 4) Cross-system collaboration, 5) Psychological, pharmacological and medical treatment,* and *6) Continued monitoring.*

**Conclusion:**

Perinatal eating disorder guideline recommendations were fairly consistent but showed considerable variability in quality and depth of recommendations. Recommendations require further contextualisation, to allow them to be operationalised and implemented within services. The review findings provide an initial framework for health care professionals responsible for supporting women with eating disorders during the perinatal period, and have several implications for policy, service delivery and health outcomes for women and their families.

**Supplementary Information:**

The online version contains supplementary material available at 10.1186/s12884-024-06995-x.

## Background

Eating disorders (EDs) are psychiatric disorders characterised by abnormal eating or weight-control behaviours [1]. Diagnostic systems recognise different diagnoses of EDs, each with unique presenting symptoms, including Anorexia Nervosa (AN), Bulimia Nervosa (BN), Binge Eating Disorder (BED), Other Specified Feeding or Eating Disorder (OSFED) and Unspecified Feeding or Eating Disorder (UFED) [2, 3]. Eating disorders considerably impair physical health, disrupt psychosocial functioning [2], and are accompanied by significantly increased mortality rates [4], with AN having a higher mortality rate than any other mental health disorder [5*].

Lifetime prevalence of EDs is estimated at between 2.58%—8.4% for women and 0.74%—2.2% for men [6, 7]. Women with a history of EDs, particularly AN, are at elevated risk of unplanned pregnancy due to the misconception that menstrual disturbance, caused by the ED, makes them unable to conceive [8]. Approximately 5% of women have an ED during pregnancy [9]. Women with EDs are more likely to experience negative feelings upon discovering that they are pregnant when compared with the general population, perhaps due to the unexpected discovery, the prospect of pregnancy-related weight gain [10] and managing the increased stress of eating for the baby [11]. Despite evidence that women are often able to reduce ED-behaviours during pregnancy for the benefit of their baby (e.g., [12, 13]) or due to having a new ‘context’ to their weight gain [14], ED symptoms and cognitions remain elevated in this group of women [15].

Literature has found that 12.8% of postpartum mothers are diagnosed with an ED, compared with 5.3% of prepartum mothers [16] suggesting that it is common for ED symptoms to return or worsen after birth irrespective of symptoms during pregnancy [14]. During the postpartum period, women often feel they no longer have an ‘excuse’ for residual weight gain and the societal pressure to maintain the slim ideal returns [17], which results in a strong desire to return to pre-pregnancy shape and weight [14]. Therefore, the literature suggests that the perinatal period (pregnancy and up to 12 months after childbirth; [18]) is a high-risk period for the development, maintenance and re-occurrence of EDs in women.

Eating disorders during the perinatal period are associated with various risks to maternal and foetal health. In terms of risks to the mother, current or past history of an ED is associated with higher levels of depression and anxiety in pregnancy and postpartum [19, 20]. Additionally, evidence suggests that maternal EDs are associated with greater pregnancy and delivery complications, such as increased risk of hyperemesis gravidurum, prolonged labour, caesarean, and induced delivery [21–23]. Maternal AN has been shown to be associated with higher rates of anaemia [24] and maternal BED has been associated with higher rates of hypertension and diabetes during pregnancy [22, 24].

Regarding increased risk to the baby, maternal AN and BN are associated with restricted foetal growth [25], pre-term delivery [26], microcephaly [27], smaller head circumference [21] and small-for-gestational age [28], whereas mothers with BED are more likely to birth infants with large-for-gestational age [22, 24]. Moreover, newborns of pregnant women with EDs are at higher risk of perinatal mortality when compared with the general population [24, 29]. Substances of abuse such as alcohol, narcotics or tobacco, which are more frequent in ED patients, may have a comorbid impact on foetal development [29].

The risks associated with perinatal EDs extend into the postpartum period and influences child cognitive, psychological and physical development [30]. Mothers with EDs can demonstrate greater concern for their child’s weight and eating habits [31] and maternal EDs are associated with restrictive feeding styles and greater eating problems in their children [32, 33]. Infants of mothers with EDs have been found to demonstrate difficulties with social understanding, poorer motor skills, planning, abstract reasoning and language development [34, 35]. Research suggests that children of mothers with EDs are at a higher risk of developing ED symptoms themselves [22, 36] and experiencing emotional and conduct problems [37, 38].

Given the increased maternal and infant-related risks associated with perinatal EDs, there is an emphasis on the clinical importance of early identification and response by skilled clinicians to mitigate these risks [39]. It is therefore imperative that high quality clinical practice guidelines (CPGs) exist to set optimum care standards for the range of health care professionals (HCPs) supporting women during the perinatal period.

Clinical practice guidelines are defined as statements that include recommendations intended to optimise patient care that are informed by a systematic review of evidence and an assessment of the benefits and harms of alternative care options [40]. Clinical practice guidelines have been considered an essential part of quality medical practice for several decades and offer a way of bridging the gap between policy, best practice, local contexts and patient choice [41]. However, CPGs can show great variety regarding their depth of evidence base and general methodological quality [42]. Systematic reviews of CPGs are becoming increasingly popular, due to their efficacy in synthesising the current knowledge, as well as identifying gaps in the knowledge about a particular issue [42].

An international review of evidence-based clinical treatment guidelines for EDs, without a focus on the perinatal period [43] found consistency amongst psychological treatments and for single medications with a larger evidence base, such as Cognitive Behavioural Therapy (CBT), self-help treatment and Selective Serotonin Reuptake Inhibitors (SSRIs; for BN and BED only). However, inconsistencies were found amongst recommendations without a large evidence base, such as psychodynamic therapy and alternative pharmacological treatments [43]. A systematic review of CPGs on nutrition in pregnancy [44] found that appropriate maternal nutrition in pregnancy is crucial but found significant heterogeneity between CPGs regarding recommended doses for vitamins, folic acid, micronutrient intake and overall gestational weight gain. Additionally, there was no guidance upon the adaptation of these recommendations for women with EDs. Individuals with EDs require a careful correction of biochemical abnormalities before weight gain [45], due to the potentially lethal risks of refeeding syndrome [46]. Therefore, general recommendations for maternal nutrition during pregnancy should not be applied to women with EDs and adapted nutritional recommendations are required. To date, there are no systematic reviews of the CPGs for EDs specifically within the perinatal period.

The current systematic review aimed to identify and synthesise CPGs containing recommendations for the assessment, management and treatment of women with EDs during the perinatal period.

## Methods

### Design

The systematic review protocol was registered with the International Prospective Register of Systematic Reviews (PROSPERO) in November 2023 (registration number CRD42023391610). The review was conducted in accordance with the Preferred Reporting Items for Systematic Reviews and Meta-Analyses (PRISMA) guidelines [47] and guided by Johnston et al.’s (2019) methodological guide for systematic reviews of CPGs [42], as these types of unique knowledge syntheses require tailored approaches.

### Search strategy

Clinical practice guidelines were identified through a systematic search of three international bibliographical databases (PubMed, CINAHL and PsycINFO) and five guideline repository databases (Turning Research into Practice Database [TRIP], Scottish Intercollegiate Guideline Network [SIGN] and International Guidelines Library [GIN], World Health Organisation [WHO] and the National Institute for Health and Care Excellence [NICE]). Grey literature was identified by hand searching relevant charities and organisations’ websites, reference lists of relevant systematic reviews and Google. Searches were completed in November 2023.

The search strategy was developed by the research team in collaboration with a university librarian, containing free text terms and Boolean operators specific to the review question and database. Search results were filtered by publication type and/or subject type (e.g., ‘clinical guidelines’, ‘practice guidelines’ or ‘guideline’) where available. See Additional File 1 for the full search strategy.

### Eligibility criteria

The ‘PICAR’ (Population, Intervention, Comparators, Attributes of eligible CPGs, Recommendation characteristics) framework was used to guide review inclusion and exclusion criteria (Additional File 2), an adaptation of the PICO (Patient/problem, Intervention/exposure, Comparison/control, Outcome) statement for systematic reviews of interventions, which has been modified to meet the specific needs of systematic reviews of CPGs [42].

Clinical practice guidelines were eligible for inclusion if they reported at least one CPG recommendation relating specifically to EDs during the perinatal period. Clinical practice guidelines for EDs **or** the perinatal period were included in full-text screening, to check for specific sections relevant to the review question (e.g. guidelines for EDs, where there was a section on the perinatal period or guidelines for the perinatal period, where there was a section on EDs).

Included records must have been clearly labelled as CPGs, guidance or guidelines. Due to the anticipated lack of guidance available for the specificity of the review question, no restrictions were made on publication date, and it was decided to utilise publication date as information to reflect upon within the synthesis and discussion. Only the latest version of the CPG was included. Due to time and resource restrictions, CPGs were required to be available in the English language, though no restrictions were made on country of publication. Only CPGs issued or endorsed by national or international scientific societies, professional colleges, charitable or not-for-profit organisations, and government organisations were included and there were no restrictions placed on intended CPG-user.

### Study selection and data extraction

Once searches were complete, all obtained records were imported into a reference management software (Rayyan; [48]) and duplicates were removed. One author completed the title and abstract/summary screening, and a second independent reviewer screened a randomised selection of 10% of the titles and abstract/summaries based on the eligibility criteria. Full texts were screened by one author.

A unique data extraction tool was designed by the first author, based on the PICAR table and research question. The data extraction tool was utilised to obtain the authors/publishing organisation, year of publication, country of publication, type of CPG (e.g. government, charitable organisation), version, topic addressed (e.g. EDs during perinatal period or broader) and any CPG recommendations relating to the assessment, management or treatment of EDs during the perinatal period.

### Quality assessment

The included records were appraised for methodological quality using the Appraisal of Guidelines, Research and Evaluation (AGREE-II) instrument, which is designed to assess the quality of practice guidelines across the spectrum of health, provide direction on guideline development, and guide what specific information ought to be reported in guidelines [47]. The AGREE-II tool is the most widely used and comprehensively validated guideline appraisal tool internationally [49].

The AGREE-II assesses 23 items across six domains; *scope and purpose*, *stakeholder involvement*, *rigour of development*, *clarity of presentation*, *applicability*, and *editorial independence*; using a 7-point Likert scale from 1 (strongly disagree) to 7 (strongly agree).

Each guideline was independently rated by two reviewers. If an item was unable to be scored due to missing information, it was rated 1. As per the AGREE-II User Manual [50], domain scores were calculated by totalling the scores of each item by each reviewer and scaling the total as a percentage of the maximum possible score. Both raters then agreed on an overall quality score for each domain from 1–7, and a decision was made upon whether the raters would recommend use of the guideline (yes, yes in conjunction with other CPGs, or no).

Due to the limited guidelines in the area, the authors decided beforehand that the quality ratings of the included guidelines would not be used to determine inclusion in the review, but to inform the reporting and conclusions of the review.

### Synthesis

A narrative synthesis [51] was used to describe and summarise the relevant CPG recommendations. Recommendations were first broadly categorised into those which focussed on assessment, management or treatment. They were then compared and contrasted to identify patterns, including an assessment of the strength of recommendations by considering the frequency of recommendations appearing across different CPGs.

## Results

**Fig. 1:**
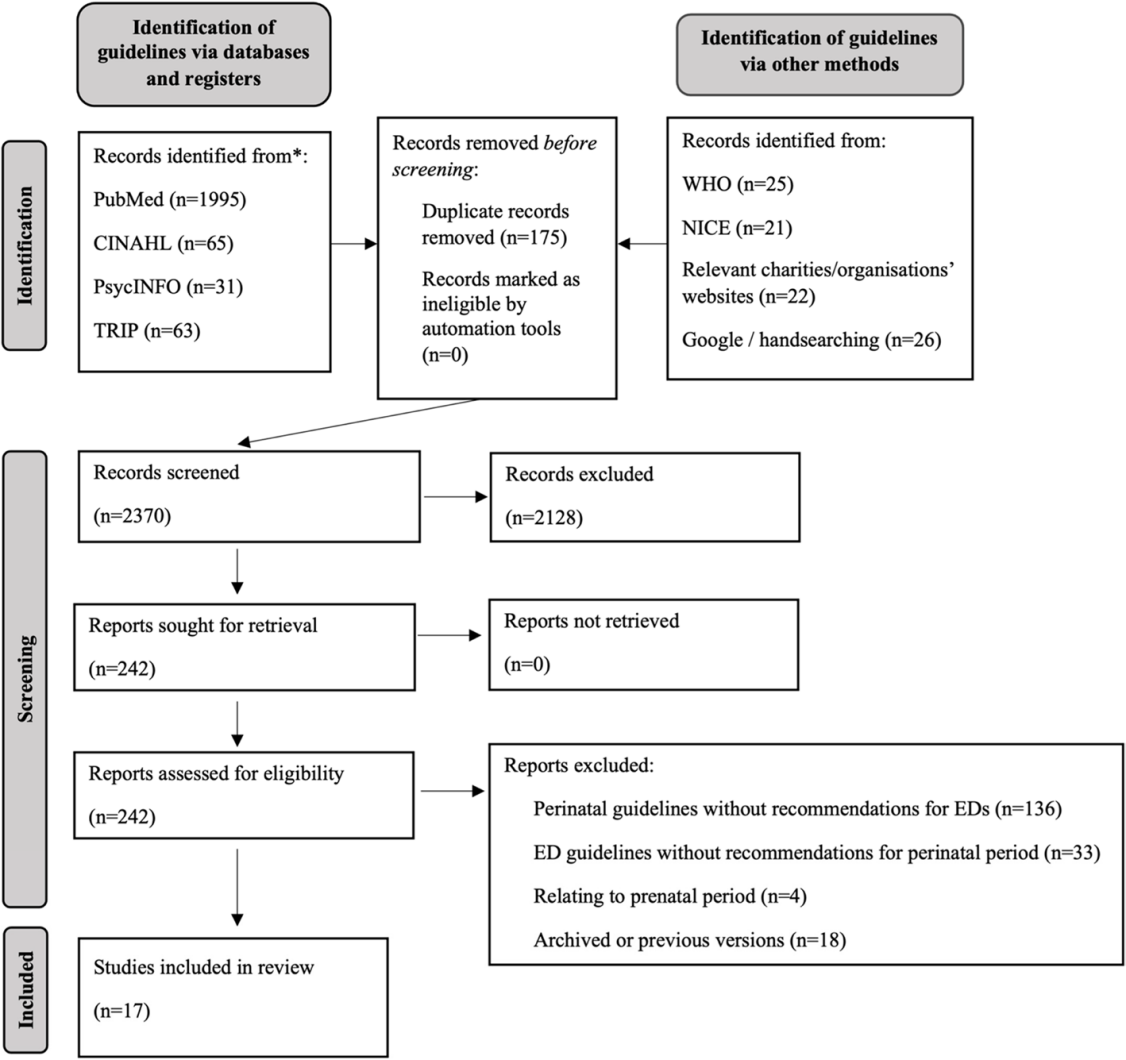
PRISMA Flow Diagram

The PRISMA flow chart (Fig. 1) illustrates the search process and outcome. The systematic search strategy initially identified a total of 2548 records. After duplicates were removed, 2373 records were screened at title/abstract level, and 2133 were removed. Inter-rater reliability of title/abstract screening was strong (*κ* = 0.83). The remaining 242 records were screened at full-text level and 17 records were eligible for inclusion in the review.

The 17 included CPGs are referred to throughout the review as acronyms of their author/organisation. See Table 1 for the included CPGs, acronyms used throughout the review and descriptive statistics, in order of publication. Guidelines were published in England (*n* = 6), Australia (*n* = 4), France (*n* = 2), Scotland (*n* = 1), Canada (*n* = 1), Germany (*n* = 1), Spain (*n* = 1) and America (*n* = 1). Of the 17 included in CPGs, 10 were ED guidelines containing perinatal recommendations, three focused specifically on EDs during the perinatal period, and four were perinatal guidelines containing ED recommendations.

**Table 1:** Descriptive information of included CPGs

	**Author/Organisation**	**Name of Guidelines**	**Acronym**	**Year of Publication**	**Country of Publication**	**Type of clinical practice guideline**
1	Royal College of Psychiatrists [52*]	Medical Emergencies in Eating Disorders: Guidance on Recognition and Management	RCP	2023	England	Professional college
2	University of Arkansas for Medical Sciences [53*]	Eating Disorders during Pregnancy and Postpartum	UAMS	2023	America	Professional college
3	World Federation of Societies of Biological Psychiatry [54]	The Pharmacological Treatment of Eating Disorders	WFSBP	2023	England	International scientific society
4	French National College of Midwives [55]	Weight Changes, Nutritional Intake, Food Contaminants, and Supplements in Women of Childbearing Age, including Pregnant Women: Guidelines for Interventions during the Perinatal Period	FNCM	2022	France	Professional college
5	Scottish Intercollegiate Guidelines Network [56*]	SIGN164: Eating Disorders	SIGN	2022	Scotland	Government organisation
6	The British Psychological Society [57*]	Good Practice Guidelines: Trainee clinical psychologists and qualified clinical psychologists working with people with eating disorders	BPS	2021	England	Scientific society
7	National Institute for Health and Care Excellence [5]	Eating Disorders: Recognition and Treatment	NICE-ED	2020	England	Government organisation
8	The Australia & New Zealand Academy for Eating Disorders [58]	Eating disorder treatment principles and general clinical practice and training standards	ANZAED	2020	Australia	Not-for-profit organisation
9	Department for Health and Wellbeing, Government of South Australia [59*]	South Australian Perinatal Practice Guideline Eating Disorders and Pregnancy	SADHW	2020	Australia	Government organisation
10	National Institute for Health and Care Excellence [60*]	Antenatal and Postnatal Mental Health: Clinical Management and Service Guidance	NICE-MH	2020	England	Government organisation
11	Society of Obstetricians and Gynaecologists of Canada [61]	Canadian guideline for physical activity throughout pregnancy	SOGC	2019	Canada	Scientific society
12	DGPM, DGESS, DGKJP, DGPPN, DKPM & DGPs* [62*]	Joint German Guideline: Diagnosis and Treatment of Eating Disorders	JGG	2018	Germany	Scientific society
13	National Eating Disorders Collaboration [63*]	Pregnancy and Eating Disorders: a Professional’s Guide to Assessment and Referral	NEDC	2015	Australia	Government organisation
14	Royal Australian and New Zealand College of Psychiatrists [64]	Clinical Practice Guidelines for the Treatment of Eating Disorders	RANZCP	2014	Australia	Professional college
15	Royal College of Obstetricians and Gynaecologists [65*]	Management of Women with Mental Health Issues during Pregnancy and the Postnatal Period	RCOG	2011	England	Professional college
16	French National Authority for Health (Haute Autorité de Santé) [66]	Clinical Practice Guidelines: Anorexia Nervosa: Management	FNAH	2010	France	Government organisation
17	Working group of the Clinical Practice Guideline for Eating Disorders, Catalan Agency for Health Technology Assessment and Research [67*]	Clinical Practice Guideline for Eating Disorders	CAHTA	2009	Spain	Not-for-profit organisation

^*^DGPM German Society for Psychosomatic Medicine and Medical Psychotherapy, *DGESS *German Society for Eating Disorders, *DGKJP *German Society for Child and Adolescent Psychiatry, Psychosomatic Medicine and Psychotherapy, *DGPPN *German Association for Psychiatry, Psychotherapy and Neurology, *DKPM *German College for Psychosomatic Medicine, *DGPs *German Psychological Society

The included CPGs were published or most recently updated between 2009 – 2023. Two records (JGG [62*] & CAHTA [67*]; See Table 1) displayed watermarks highlighting that the guidelines were due to be updated, advising caution. The author contacted both authors to enquire about revised guidelines and no response was received. These guidelines were still included in the review due to the eligibility criteria.

### Quality appraisal

The quality assessment for the 17 included CPGs is illustrated in Table 2. The author sought additional information upon the guideline development process from the authors of two CPGs, as noted within Table 2. Individual AGREE-II item scores per guideline are provided in Additional File 3.

**Table 2:** AGREE-II quality appraisal scores

		**Domain 1: Scope and Purpose**	**Domain 2: Stakeholder Involvement**	**Domain 3: Rigor of Development**	**Domain 4: Clarity of Presentation**	**Domain 5: Applicability**	**Domain 6: Editorial Independence**	**Overall Guideline Assessment**	**Overall Guideline Assessment**
**Organisation/Author(s) and Year**									
1	Royal College of Psychiatrists (2023)	100%	100%	100%	100%	100%	75%	7	YES
2	University of Arkansas for Medical Sciences (2023)^a^	56%	22%	36%	81%	25%	25%	3	YES – in conjunction with other CPGs
3	World Federation of Societies of Biological Psychiatry (2023)	89%	33%	64%	47%	4%	50%	4	YES – in conjunction with other CPGs
4	French National College of Midwives (2022)	100%	83%	85%	100%	0%	50%	5	YES
5	Scottish Intercollegiate Guidelines Network (2022)	100%	100%	97%	100%	88%	79%	7	YES
6	The British Psychological Society (2021)	92%	81%	33%	86%	8%	0%	4	YES – in conjunction with other CPGs
7	National Institute for Health and Care Excellence (2020)	100%	100%	88%	100%	100%	100%	7	YES
8	The Australia & New Zealand Academy for Eating Disorders (2020)	97%	100%	48%	100%	56%	75%	6	YES
9	Department for Health and Wellbeing, Government of South Australia (2020)	100%	56%	10%	67%	0%	0%	3	YES – in conjunction with other CPGs
10	National Institute for Health and Care Excellence (2020)	100%	100%	74%	97%	100%	100%	7	YES
11	Society of Obstetricians and Gynaecologists of Canada (2019)	100%	100%	75%	100%	73%	50%	6	YES
12	DGPM, DGESS, DGKJP, DGPPN, DKPM & DGPs (2018)	61%	78%	88%	72%	42%	33%	5	YES
13	National Eating Disorders Collaboration (2015)^a^	78%	78%	6%	100%	21%	0%	4	YES – in conjunction with other CPGs
14	Royal Australian and New Zealand College of Psychiatrists (2014)	100%	61%	92%	100%	6%	100%	6	YES
15	Royal College of Obstetricians and Gynaecologists (2011)	83%	6%	38%	100%	25%	8%	4	YES – in conjunction with other CPGs
16	French National Authority for Health (Haute Autorité de Santé, 2010)	100%	83%	72%	83%	46%	13%	5	YES
17	Working group of the Clinical Practice Guidelines for Eating Disorders, Catalan Agency for Health Technology Assessment and Research (2009)	100%	67%	79%	100%	56%	100%	6	YES

^a^These CPGs were quality appraised using additional information provided by the author

To measure inter-rater reliability between AGREE-II scores for each CPG, a weighted kappa was calculated for each CPG using SPSS Version 29.0 [68] due to the data being ordinal. The weighted kappa values ranged from moderate κ_w_ = 0.537 (95% CI 0.079 to 0.994) to perfect κ_w_ = 1.00 (95% CI 1.00 to 1.00). Overall inter-rater reliability across all CPGs was almost perfect with an intra-class correlation of 0.989 (95% CI 0.948 to 0.997).

### Recommendations

**Fig. 2:**
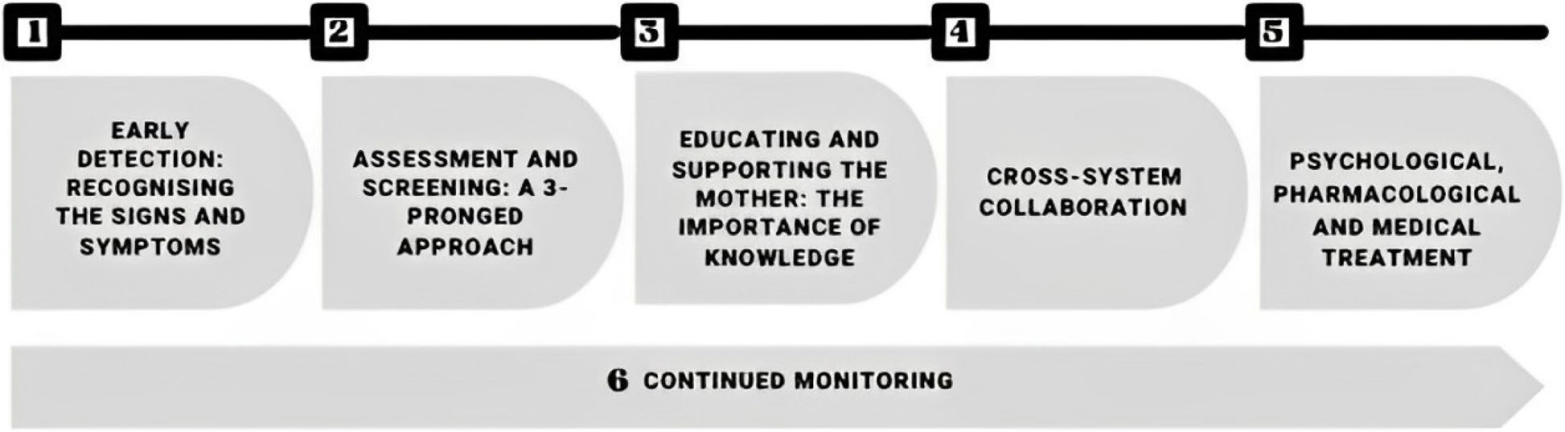
Model of Recommendations

Recommendations for the assessment, management and treatment of EDs during the perinatal period were extracted from the 17 included CPGs, and synthesised into six overall recommendations which are summarised in Fig. 2.

Table 3 provides a summary of the recommendations, which are described in further detail below.

**Table 3:** Summary of recommendations

Recommendation	Brief Description
1: The Importance of Early Detection—Recognising the Signs and Symptoms	Screening and recognition of possible EDs as early as possible within perinatal appointments. HCPs should be aware of possible signs and symptoms, both in general and specific to the perinatal period
2: Assessment and Screening—A Three-Pronged Approach	In-depth assessment of possible EDs during pregnancy and postpartum, including a validated screening tool, information screening questions and (if indicated) a physical examination
3: Educating and Supporting the Mother—The Importance of Knowledge	Ensuring women are provided with adequate information about how their ED symptoms may change, recommended nutrition during pregnancy and postpartum, feeding the baby and supported with coping skills. HCPs should have training in identifying and managing patients with EDs and the risks to mother and baby
4: Cross-System Collaboration	Liaison between all systems and professionals involved in the care of women, including collaborative care planning, shared management plans and provision of regular updates. Onward referrals to other specialist services is recommended when appropriate
5: Psychological, Pharmacological and Medical Treatment	ED treatments should be adapted to meet the additional needs of pregnant and postpartum women. Psychological treatment should follow NICE ED guidelines. Certain pharmacological treatments are advised against during pregnancy. Nutritional rehabilitation is recommended, and women should be hospitalised if physical examinations identify risks
6: Continued Monitoring	Enhanced and ongoing monitoring of maternal and foetal health throughout pregnancy and postpartum, including ongoing foetal growth scans and maternal health and blood checks (where there are concerns about maternal weight and/or nutritional intake). During the postnatal period, monitor parent–child relationship and maternal attitude towards motherhood

#### Recommendation 1: the importance of early detection—recognising the signs and symptoms

The importance of early detection of EDs within pregnant women was highlighted within four CPG documents [53*, 56*, 59*, 63*]. Both UAMS [51] and NEDC [63*] recommended screening during the initial pre-natal visit to allow for early intervention, though it was noted that screening for EDs can take place during other perinatal appointments, including the foetal ultrasounds, the hospital admission interview or the third trimester check-up. SIGN [56*] recommend that HCPs should routinely and sensitively enquire about EDs and previous EDs throughout the pregnancy and postnatal period, whilst also being aware of the potential barriers for disclosure. See further recommendations regarding assessment and screening in Recommendation 2.

SADHW [59*] and UAMS [53*] highlight the cruciality of relevant HCPs being knowledgeable about and sensitive to the possible signs and symptoms which may indicate an ED specifically within expectant or new mothers. NEDC [63*] note that ED signs and symptoms can manifest as normal symptoms of pregnancy, such as tiredness, or disguised as pregnancy-related ailments, such as morning sickness disguising self-induced purging. Therefore, it is important that HCPs working with women during the perinatal period are aware of potential indicators of an ED within this population. UAMS [53*] provide a list of ‘red flags’ for perinatal EDs and NEDC [63*] provide a list of signs and symptoms of EDs specific to pregnancy and postpartum (Table 4).

**Table 4:** Signs and Symptoms of Perinatal EDs

CPG	Signs and Symptoms
UAMS	• Severely over- or underweight (BMI > 35 or < 17) • Resistance to getting on a scale • Fear of weight gain in pregnancy • Lack of weight gain over two consecutive prenatal visits in the second trimester • Attendance to a medical weight loss clinic, especially if patient is not significantly overweight • History of infertility and/or menstrual irregularity • Hyperemesis gravidarum • Gastrointestinal problems • Low bone density • Laxative dependence • Oral mucosa damage or dental problems • Persistent electrolyte abnormalities • Presence of a mood or anxiety disorder • Stimulant use in adulthood
NEDC	**Specific to pregnancy:** • Little or no weight loss (in the case of binge-eating disorder for example) or weight gain (in the case of anorexia nervosa for example) over the course of the pregnancy, despite a growing foetus • Problems with foetal growth and development • Gestational diabetes • Respiratory problems • Miscarriage • Premature labour / preterm deliver • Complications during labour • Unplanned caesarean • Low birth weight • Stillbirth or foetal death • Postnatal depression **Specific to postnatal/new mothers:** • A history of eating disorders prior to pregnancy or during pregnancy • Postnatal depression • Rapid, otherwise unexplained postnatal weight loss or weight gain • Negative feelings towards the baby or to becoming a mother • Anxiety about baby’s appearance – e.g. overly referring to the baby as ‘chubby’ • A strong focus on pre-baby shape and/or returning to body shape-inspired exercise soon after childbirth • Compulsive/obsessive breast-feeding (can be associated with a desire to lose weight quickly) • Difficulty maintaining or loss of milk supply • Signs associated with purge activities such as signs of excessive vomiting (bad breath, eroding teeth), laxative abuse, calluses on knuckles (from forced purging) • Irregular weight gain in the infant • Signs of under or over feeding in the infant • Signs of malnutrition or under-nutrition in the mother and infant

#### Recommendation 2: assessment and screening—a three-pronged approach

Clinical practice guidelines highlighted a need for an in-depth, three-pronged approach to assessing EDs during the perinatal period, including 1) the use of a validated screening questionnaire [53*, 59*, 63*], 2) informal screening questions [56*, 59*, 63*] and 3) if indicated, a physical examination [59*, 63*].

### Validated screening questionnaires

UAMS [53*] note that there are multiple screening questionnaires for EDs available; however, none are specific to the perinatal period. Most recommended [53*, 59*, 63*] is the SCOFF Questionnaire [69]. The SCOFF is a simple, five question screening tool designed to raise the suspicion that an ED might exist before rigorous clinical assessment. The five questions are as follows with each ‘yes’ indicating one point, and a score of two or more indicates a likely diagnosis of AN or BN:

- Do you forcibly make yourself Sick because you feel uncomfortably full? YES/NO
- Do you feel like you lose Control when you are eating? YES/NO
- Have you recently lost more than One stone (14 lb) in a 3-month period? YES/NO
- Do you believe yourself to be Fat when others say you are too thin? YES/NO
- Would you say Food dominates your life? YES/NO

### Informal screening questions

FNCM [55], SIGN [56*], SADHW [59*] and NEDC [63*] also recommend the use of additional informal questioning to detect the possible presence of an ED. SIGN [56*] recommend that during pregnancy and the postnatal period, HCPs should routinely, and sensitively, enquire if the woman has a current or past history of ED. NEDC [63*] recommend the following informal screening questions:

- Does your weight affect the way you feel about yourself?
- Are you satisfied with your eating patterns?
- Are you dieting or trying to lose weight?
- Do you think you have an eating problem?
- Do you worry a lot about your weight?
- Do you worry a lot about your body shape? How do you feel about the changes happening to your body?
- Is there a history of eating disorders in your family?
- Do you have prior experience of an eating disorder?
- Is there a history of depression or anxiety in your family, or have you ever suffered from depression or anxiety?
- Do you have any other illnesses, such as Polycystic Ovarian Syndrome or Diabetes?
- (Postnatal) Are you satisfied with how your baby is gaining weight?

### Physical examination

For women with a history of AN, SIGN [54] recommend enhanced screening for iron deficiency anaemia. SADHW [57*] and NEDC [61] recommend a more extensive physical examination if the screening questionnaire and informal questioning indicate a possible ED. The following physical checks are recommended (62):

- General physical state (well vs. unwell)
- Body temperature (< 36 °C)
- Pulse rate for resting and standing (< 60 bpm, regular or irregular)
- Blood pressure lying and standing (postural drop > 20 mmHg)
- Alertness vs. somnolence/sleepiness
- Height and weight history and weight/height proportion – preconception, during pregnancy and postnatal
- Menstruation pattern/history
- Hydration (tongue, lips, sunken eyes, skin)
- Signs of vomiting (ketones on breath, bad breath, eroded teeth)
- Fundal measurements according to individual’s expected progression of foetal growth (in pregnancy)
- Deep irregular sighing; breathing seen in ketoacidosis
- Peripheral circulation (limbs, extremities) and cold peripheries
- Physical changes, such as swelling in cheeks, jaw, ankles; calluses on knuckles; abdomen scaphoid
- Electrolyte disturbances (thirst, dizziness, fluid retention, swelling, weakness/ lethargy, muscle twitches)
- Alkaline urinary pH

UAMS [53*] recommend a physical examination along with obtaining the following laboratory tests, if there is evidence of a past or present ED:

- Complete Blood Count (CBC) blood test
- Basic metabolic panel
- Amylase
- Lipase
- Iron levels
- Magnesium
- Phosphate
- Vitamin D
- Obtain electrocardiogram (ECG)

#### Recommendation 3: educating and supporting the mother—the importance of knowledge

Guidelines recommend taking a collaborative approach to providing support and education to expectant or new mothers with EDs [5, 56*, 59*, 60*, 63*]. SIGN [56] recommend that HCPs inform pregnant women with EDs how their symptoms may change during the pregnancy and the postnatal period. These discussions should involve counselling on the balance of untreated EDs and medication exposure during the different stages of the perinatal period, allowing women to make informed choices.

Nutritional support and education is also recommended during pregnancy and postpartum. NICE-ED [5], SADHW [59*], NICE-MH [60*] and NEDC [63*] recommend educating women on healthy nutrition during pregnancy and its relationship to foetal development and their own mental health. Postnatally, it is recommended that HCPs provide education around feeding the baby [5, 60*], including the provision of breastfeeding support [59*, 63*]. No CPGs provided additional advice for supporting women with EDs to breastfeed. This education should be provided to support women to make their own decisions about feeding and breastfeeding [52*]. NICE-ED [5] recommend utilising the NICE guidance on maternal and child nutrition [70] to inform these discussions.

NEDC [63*] recommend the provision of advice and guidance to improve coping skills as well as self-esteem and confidence in expecting and new mothers, which should include encouraging acceptance of body image and the normalisation of physical changes [59*].

In order to implement this recommendation, it is important that healthcare professionals working with pregnant and postnatal women have training, relevant to their role, in identifying and appropriately managing patients with EDs [56*]. Health care professionals should be alert to the potential physical, psychological and social risks associated with EDs [57*, 63*], such as increased risk of obstetric complications [56*] and social isolation [63*]. Psychological professionals should be aware of the comorbid risk of diabetes in perinatal EDs, the medication and differing dietary requirements [57*]. There should also be an awareness that cultural practices or pregnancy diets in minority ethnic communities may mask EDs [56*]. Postnatally, HCPs should be aware of the possible risk of relapse and should be able to look for signs of postnatal depression or anxiety [56*, 63*], indicating a need for HCPs to have basic mental health training.

Furthermore, HCPs should be aware of increased risk of impaired mother-infant interactions postnatally [59*], with an ability to identify any negative emotions to the infant and anxious or avoidant attachment patterns [63*].

#### Recommendation 4: cross-system collaboration

The most highly recommended course of action when working with women with EDs during the perinatal period is the collaboration and liaison between the various systems and professionals involved [53*, 56*, 57*, 59*, 63*, 66*], including midwives, obstetricians, ED specialists, perinatal mental health services, general practitioners, health visitors, psychologists, dieticians, family or early childhood nurses and other agencies if relevant such as children’s services [56*, 57*, 59*, 63*]. A discussion with the mother is recommended prior to notifying antenatal services about the ED [63]. Furthermore, NEDC [63*] recommend the engagement of the wider system, such as family members, carers, partners and friends, to provide support and help, with the women’s permission.

The purpose of this cross-system collaboration is to communicate regularly [63*], liaise around care planning, share management plans [59*], update other clinicians with relevant information, monitor risks to the mother and child [63*] and, where the mother has AN, to ensure the foetus is developing normally [66*].

An important part of this cross-system collaboration involves appropriate referrals to other services or specialists. A referral to ED services and/or perinatal mental health services for pregnant women with a current or past ED is recommended [55, 57*, 59*, 63*, 65*], as well as general psychiatric services to manage any distress or serious illness secondary to the ED, such as psychosis, suicidal ideation, self-neglect, evidence of harm to others or significant interference with daily functioning [53*, 65*]. A referral to a dietician experienced in ED management is also recommended for women with nutritional management difficulties or significant laboratory/vital abnormalities [53*, 58]. Furthermore, NICE-ED [5*] recommend a nominated professional to oversee the monitoring and support of pregnant women with EDs.

#### Recommendation 5: psychological, pharmacological and medical treatment

It is recommended that all treatment for perinatal EDs should be adapted to meet the individual needs of the patient and their family, as well as covering the postnatal period [57*]. Treatment goals are recommended to be manageable and realistic [57*] as full recovery may feel overwhelming when accompanied by all the physical and emotional changes associated with pregnancy. If a pregnant person with ED is already receiving psychiatric treatment, it is recommended that the HCP assesses the efficacy and safety of the current treatment during pregnancy [53*].

NEDC [63*] recommend a referral to a hospital or emergency room if the mother’s or baby’s life may be at risk. For those requiring an inpatient admission, it is recommended that both the wellbeing of the mother and the baby are accommodated [57*].

### Psychological intervention

Psychological intervention for perinatal EDs was recommended by three CPGs [5*, 53*, 60*]. NICE-ED [5*] and NICE-MH [60*] recommend that women with EDs in the perinatal period are offered treatment in line with the NICE guidelines for treating EDs generally. For the psychological treatment of AN, NICE recommend either individual ED-focussed cognitive behavioural therapy (CBT-ED), Maudsley Anorexia Nervosa Treatment for Adults (MANTRA) or specialist supportive clinical management (SSCM). For the psychological treatment of BN, NICE recommend bulimia nervosa-focussed self-help, cognitive behavioural self-help materials supplemented with brief supportive sessions or CBT-ED. For BED, NICE recommend a BED-focussed self-help programme or CBT-ED.

### Pharmacological intervention

UAMS [53*] recommended the use of Fluoxetine, Citalopram and Sertraline and Olanzapine, for pregnant women with EDs. Topiramate was not recommended due to reproductive safety concerns [53*, 54] and Fluvaxamine was not recommended due to the lack of current research [53*]. For patients who are laxative dependent, UAMS [53*] recommend against the use of osmotic or stimulant laxatives during pregnancy due to their increased risk of dehydration and electrolyte imbalance.

### Medical intervention

UAMS [53*] recommend nutritional rehabilitation, including behavioural management, nutritional counselling and medical monitoring for the treatment of EDs in the perinatal period. The nutritional monitoring should specifically focus on iron, vitamin B12, thiamine and folic acid, and vitamin supplementation should be offered if indicated. UAMS [53*] also recommend the monitoring of caffeine intake, noting that patients with EDs tend to over-consume caffeine.

UAMS [53*] provides a flowchart for the subsequent course of medical action for women with perinatal EDs following physical examinations. It is recommended that women should be hospitalised if their heart rate is below 30, there is a presence of tachycardia or hypotension, core temperate is less than 35 degrees Celsius, BMI is less than 16 kg per metre squared, corrected QT internal of greater than 0.499 ms, presence of liver failure, pancreatitis or severe electrolyte disturbance.

It is also recommended that people with EDs should discuss the advantages and disadvantages of moderate to vigorous intensity physical activity with their obstetric care provider prior to participation [61].

#### Recommendation 6: continued monitoring

Guidelines consistently recommend enhanced and ongoing monitoring of the maternal and foetal health throughout pregnancy and the postnatal period to ensure good health for both mother and baby [5*, 59*, 60*, 62*, 63*, 66*, 67*].

### Pregnancy

In pregnancy, where there are concerns about weight and/or nutritional intake, ongoing foetal growth scans and maternal weight monitoring are recommended [56*, 59*], as well as regular checks of vital signs (heart rate, respiratory rate, blood pressure and body temperature) and basic metabolic panel; a blood test that measures eight different substances in the blood [53*].

### Postpartum

Monitoring should continue into the postpartum period due to the risk of relapse [56*], the risk of postpartum depression increasing [62*] and, where the woman has AN, to avoid deterioration in mother’s nutritional or mental condition [66*]. This should include exploring any changes in the mother’s attitudes towards eating and body shape [63*], which may indicate increased risk.

There should be importance placed on the monitoring of parent–child interactions, due to increased risk of difficulties within mother-infant interactions and to ensure that the mother’s concerns are not transferred to the infant [59*] This includes monitoring parenting skills, parent–child relationship, attachment patterns and feelings towards motherhood in general [63*, 66].

## Discussion

A systematic review of peer-reviewed and grey literature identified 17 CPGs containing recommendations for the assessment, management and treatment of EDs during the perinatal period. These CPGs were appraised for quality using the AGREE-II tool [50] and synthesised into six overall recommendations using narrative synthesis [51].

There was disparity between the quality of the 17 included CPGs, ranging from scores of three out of seven (low quality), to seven out of seven (high quality). There was a high level of agreement amongst the recommendations between CPGs, demonstrating the strength of the overall recommendations.

It is important to consider the potential barriers implementing six recommendations identified in this review. Though the current review emphasises the importance of early detection and screening of perinatal EDs, there is a large body of evidence indicating high levels of shame and secrecy in women with EDs, which may pose a barrier to the application of this recommendation (e.g., [71, 72]). For example, women may not view their eating behaviours as problematic or may be more sensitive to judgement than others, leading to denial or reluctance to disclose symptoms [72]. Women with EDs are more likely to perceive the external world as more negative and critical in the postpartum period, than women without EDs [73]. Communication challenges are common when working with individuals with EDs [74] and women are hesitant to disclose EDs during the perinatal period due to high levels of shame and secrecy, meaning careful open-ended questioning, sensitive communication and vigilance by HCPs is important [72]. This could be supported by implementing Sect. "Search strategy" of the recommendations, which offers suggestions for informal screening questions, and upskilling HCPs (recommendation 3) as improving the ED health literacy of HCPs is an integral part of improving disclosure of an ED during pregnancy [14]. Additionally, it is thought that continued monitoring (recommendation 6) will support HCPs to maintain vigilance and may encourage women to disclose EDs as the mother-professional relationship develops. Misattuned professional support has been highlighted as a problematic barrier to recovery from perinatal EDs [75], and so implementing the aforementioned recommendations will provide ongoing opportunities for the disclosure and/or identification of perinatal EDs where the woman battles with shame.

Another barrier to implementing the recommendations synthesised within the current review is the lack of specificity for which HCPs are responsible for implementation. For example, the guidance does not specify which professionals should be responsible for the physical examination, which could be a barrier if the HCPs in contact with the expecting or new mother do not perceive themselves to be responsible for this aspect, or do not feel confident in assessing the aforementioned factors. Health care professionals working with patients with EDs commonly report limited knowledge and training and increased feelings of frustration, helplessness, incompetence and emotional exhaustion [76, 77]. Additionally, both midwives and health visitors have described a lack of opportunity and time to enquire about EDs within routine antenatal appointments [76]. Implementing recommendation 3 would ensure HCPs have role-appropriate training in identifying and managing patients with EDs, which may increase HCP confidence and consequently reduce emotional distress. However, system level barriers remain and addressing these difficulties could promote the identification of EDs in the perinatal period [76].

A systematic review exploring the implementation of perinatal mental health care in health and social care settings found barriers at the organisational level, including unclear or complex referral pathways, the scarcity of appropriate services for onwards referrals and lack of funding [78]. This highlights a need for revised national or service level policies and referral pathways, alongside appropriate funding considerations, in order to operationalise the recommendations within the current review.

The Perinatal Mental Health Care Pathways guidance [17], an NHS England document which sets out the policy initiatives and strategic context for transforming perinatal mental health care, outlines that women with complex or severe perinatal mental health problems should be referred to the specialist community perinatal mental health team by primary or secondary care services, maternity services or health visitors. Where women are already under a mental health or ED service prior to becoming pregnant, their named clinician would be in an ideal position to inform perinatal services about the woman’s difficulties upon discovering their pregnancy, to ensure appropriate onward referrals are made. Women who are not already cared for by mental health or ED services prior to their pregnancy are at greater risk of their ED being missed by perinatal services, which highlights the importance of recommendations 1 (early detection) and 2 (thorough, three-pronged assessment) being implemented by HCPs who have early contact with pregnant women, which is generally the health visitor in England. Recommendations 3 (educating and supporting the mother), 4 (cross-system collaboration) and 6 (continued monitoring) should be implemented by all HCPs within women’s pregnancy journey. Recommendation 5 (psychological, pharmacological and medical treatment) is likely to be more relevant to specialist services with the provision for treatment.

Whilst medication is not currently recommended as the sole treatment for EDs [5], medication is commonly prescribed as a supplement to other therapeutic interventions to treat comorbid psychiatric difficulties [79]. Recommendation 6.2 details which pharmacological treatments are currently recommended within the treatment of EDs during the perinatal period, and those which are recommended against due to concerns relating to reproductive safety, pregnancy-related risks and lack of research. It is noted that the CPGs did not consider how using medication to treat EDs during the perinatal period might impact on breastfeeding, which identifies a gap for further research. Current literature shows limited efficacy of pharmacological intervention in the treatment of AN [79]. Specific psychological therapies are the first-line treatment for all EDs [80]. The current review found no clinical practice recommendations for the psychological treatment of EDs which were adapted specifically for the perinatal period, despite the suggestion that pregnancy is an optimum moment for women to make lasting changes to eating and weight control practices [81]. More research is required to investigate if the benefits of psychological intervention for EDs during pregnancy extend beyond the perinatal period.

A study exploring how women with EDs experienced pregnancy, found that relapse during pregnancy held greater psychological complexity than just body and weight related distress [82], highlighting a need to tailor ED treatment during pregnancy. One author recommends utilising women’s increased motivation to recover from their ED during pregnancy for the benefit of their baby, by the development of a programme aiming to support women’s understanding of the risks their ED poses to the children and to highlight the positive effects in terms of child development and bonding [14]. Similarly, a study exploring women’s engagement with perinatal mental health services suggests that focusing on the infant and mother-infant relationship, rather than the mental health difficulty itself, can be helpful in reducing the stigma women with perinatal mental health difficulties experience [83]. It is also suggested that treatments may need to focus more on interventions that provide resources for self-nurturing, reducing anxiety, feeling out of control, and coping with stress [14], due to perinatal mental health risk factors including idealistic expectations of motherhood, a lack of social support and life stress [83]. Therefore, the literature suggests that treatments for EDs during the perinatal period should be adapted to match the woman’s new context, highlighting an area for future research to inform CPG recommendations. There is emerging literature around the efficacy of adapting ED treatments to address specific challenges associated with life transitions, such as emerging adulthood [84]. However, there remains a need for further research into mental health treatments during the transition to motherhood [85].

### Limitations

It is noted that only CPGs published in the English language were considered, due to time and resource constraints. Fourteen results were excluded from initial searches as they were not available in English, which suggests that relevant CPGs may have been available in other languages and have not been included in the current review. Although the current review included CPGs from seven different countries, inclusion of non-English CPGs may have allowed for further understanding and comparison of cross-cultural recommendations. It is also noted that the included CPGs are all published from high-income countries, meaning they may lack generalisability to lower income countries and different cultural backgrounds.

A recent review of cultural considerations in the treatment of EDs highlighted the importance of considering culture within the treatment; for example, by establishing an individualised understanding of patients within their cultural context, and the use of culturally-sensitive interventions [86]. Although the current systematic review did not yield culturally-specific recommendations, it is noted that CBT-ED (within recommendation 6.1), focuses on individualised case formulations to identify factors specific to personal and sociocultural experiences that contribute to EDs [87] and can therefore consider relevant cultural factors.

The CPGs within this systematic review did not offer guidance beyond cisgender birthing people, with no mention across the 17 CPGs of adapting recommendations for non-binary or transgender parents. It is increasingly recognized that trans and non-binary people are becoming parents; yet invisibility continues in policy, with language of maternal mental health and paternal mental health reflecting cis-heteronormativity [88].

Many of the obtained CPG recommendations do not specify which ED-diagnosis they relate to and hold a weight-centric view of EDs, assuming that the patient with an ED is underweight. Eating disorders in patients who are of a higher weight are increasingly prevalent but are consistently under-recognised and under-treated [88]. The guidelines included in this systematic review therefore lack inclusivity of the different ED diagnoses and symptoms. The author notes the lack of recommendations specific to BED in the perinatal period, a diagnosis with considerably different behavioural and physical symptoms to AN and BN. For example, the use of the SCOFF questionnaire was recommended in the current review. Despite strengths such as its simplicity and efficiency, meaning it can be administered by non-ED specialists [69], the SCOFF questionnaire demonstrates lower sensitivity to those with diagnoses of BED [89]. This could lead to false negatives in those who are overweight or obese [90]. The evidence base for the treatment of BED remains limited [91] and the quality of evidence for binge-eating outcomes is generally very low [92]. Women with BED are more likely to experience continuation of symptoms during pregnancy, than women with AN or BN, who are more likely to show a reduction of symptoms [12]. Furthermore, new onsets of BED are common during pregnancy [93], suggesting pregnancy as a window of vulnerability for BED, rather than a window of opportunity. It is therefore essential that women with BED are identified and supported during the perinatal period. The lack of sensitivity to BED within the current CPG recommendations, along with the lack of evidence for BED treatment in general, presents a need for further research and clinical development. It is important that CPGs for assessing, managing and treating perinatal EDs consider the differential behavioural and physical symptoms associated with BED, and how this might impact on the clinical care recommendations.

The current review utilised the AGREE-II tool to appraise the quality of included CPGs. Although the AGREE-II tool is the most widely used standardised tool for appraising guidelines, it has been criticised for being subjective due to lacking detailed guidance on how to perform the two overall quality assessments [49] and because it does not require users to explicitly state their reasons for supporting their judgments [94]. Despite this, inter-rater agreement between quality appraisal scores in the current review was high. Items on the AGREE-II tool were assigned scores of one where information was missing. It is acknowledged that the quality appraisal scores therefore may not accurately capture the full picture, because the guideline authors might have undertaken the required processes to meet AGREE-II item criteria, but not captured these in their published documents.

Quality scores for domain 3 (rigour of development), domain 5 (applicability) and domain 6 (editorial independence) showed considerable variability. Domain 5 scored the lowest on average. This indicates that the included CPGs may not present adequate consideration of the facilitators and barriers to the application of recommendations, advice/tools on how the recommendations can be put into practice, potential resource implications or criteria to monitor/audit the recommendations in clinical practice. Clinical practice guidelines could be improved by considering the aforementioned barriers to their implementation and providing further recommendations on how to manage these.

### Strengths

The current systematic review is the first to synthesise CPGs specifically for women with EDs during the perinatal period. An extensive search strategy was employed, and records were identified from a variety of sources. Databases and guideline repositories in the field of medicine, health care and psychology were selected, with the review question in mind, to maximise the probability of finding relevant records. As many CPGs may only be published in non-commercial or proprietary formats [42], an extensive search of the grey literature was required to identify appropriate records. The researcher employed various mechanisms by which to do this, including searching the websites of relevant charities, contacting researchers who have published literature into perinatal EDs, and delving deep into Google search returns. Whilst it is recognised that this is a manual approach with lower replicability than other search strategies, it was thought to be essential for the current systematic review and yielded records which would have otherwise been missed.

There were overarching strengths in reporting of guideline objectives and purposes and the language, structure, and format of the guidelines, with most records scoring highly in domain 1 (scope and purpose) and domain 4 (clarity of presentation). This suggests that the included CPGs show high levels of accessibility to a range of audiences, which is particularly important for the current review due to the range of HCPs responsible for implementing the recommendations (see recommendation 4).

The recommendations synthesised from the current systematic review demonstrate current knowledge and best-practice for the assessment, management and treatment of perinatal EDs across seven different countries. Implementing the provided recommendations will provide a more consistent and evidence-based package of care for women struggling with an ED during the difficult and vulnerable periods of pregnancy and postpartum. Ensuring that people with perinatal EDs receive the support they require will consequently improve the physical and psychological health of women and their babies.

## Conclusions

This review was the first to synthesise CPG recommendations for the assessment, management and treatment of EDs during the perinatal period. The systematic search led to the identification of 17 CPGs containing at least one recommendation relating to the review question, from a total of seven different countries, which were synthesised into six overall key recommendations. The breadth and quality of the CPGs varied; however, the key recommendations were fairly consistent and organised into six key recommendations: *1) early detection: recognising the signs and symptoms, 2) assessment and screening: a three-pronged approach, 3) educating and supporting the mother: the importance of knowledge, 4) cross-system collaboration, 5) psychological, pharmacological and medical treatment* and *6) continued monitoring throughout.*

The current review has several implications for policy, service delivery and health outcomes for pregnant or mothers with EDs and their babies. It is expected that the recommendations will be of particular value to HCPs working with women in the perinatal period, including midwives, health visitors, gynaecologists and general practitioners, as well as HCPs which women with EDs during the perinatal period may be referred to, such as psychiatrists, psychologists and dieticians. Implementing the provided recommendations will ensure these women are provided with an appropriate level of support and potentially reduce risk of harm to both the mother and baby. The review findings may be used as a framework for service evaluation and auditing, though further research is recommended to refine the recommendations further. In this way, the recommendations provided within the current review, alongside the current NHS focus on improving perinatal mental health, can support a move towards improving the care provided to women with EDs during the perinatal period, both at an individual HCP level and a service level.

## Supplementary Information

Supplementary Material 1.

Supplementary Material 2.

Supplementary Material 3.

## Data Availability

No datasets were generated or analysed during the current study.

## Abbreviations

ED: Eating disorder
AN: Anorexia nervosa
BN: Bulimia nervosa
BED: Binge eating disorder
OFSED: Other specified feeding or eating disorder
UFED: Unspecified feeding or eating disorder
CPG: Clinical practice guideline
HCP: Health care professional
TRIP: Turning Research into Practice Database
SIGN: Scottish Intercollegiate Guideline Network
GIN: International Guidelines Library
WHO: World Health Organisation
NICE: National Institute for Health and Care Excellence
AGREE-II: Appraisal of Guidelines for Research & Evaluation Instrument
