# Supplementary material for: A systematic review of the clinical practice guidelines for the assessment, management and treatment of eating disorders during the perinatal period
Source: BMC Pregnancy Childbirth. 2025 Jan 28;25:82. doi: 10.1186/s12884-024-06995-x (PMC11773850; doi:10.1186/s12884-024-06995-x)
Supplement: Supplementary file 1 — Supplementary Material 1. [file 12884_2024_6995_MOESM1_ESM.docx]

**Additional File 1**

*Search Strategy*

| **Database / Search Method** | **Search Strategy** |
| --- | --- |
| PubMed | Guideline OR standard OR best practice OR recommendation OR pathway OR policy  AND  Assessment OR recognition OR management OR treatment OR intervention  AND  Perinatal OR pregnan* OR postnatal OR postpartum OR antenatal OR eating disorder OR anorexi* OR bulimi* OR binge eating disorder OR ARFID OR OSFED OR UFED OR PICA OR rumination disorder  Filters: Guidelines  Restrict to: Abstract/Title |
| CINAHL | Guideline OR standard OR best practice OR recommendation OR pathway OR policy  AND  Assessment OR recognition OR management OR treatment OR intervention  AND  Perinatal OR pregnan* OR postnatal OR postpartum OR antenatal OR eating disorder OR anorexi* OR bulimi* OR binge eating disorder OR ARFID OR OSFED OR UFED OR PICA OR rumination disorder  Filters: Practice Guidelines  Restrict to: Title |
| PsycINFO | Guideline OR standard OR best practice OR recommendation OR pathway OR policy  AND  Assessment OR recognition OR management OR treatment OR intervention  AND  Perinatal OR pregnan* OR postnatal OR postpartum OR antenatal OR eating disorder OR anorexi* OR bulimi* OR binge eating disorder OR ARFID OR OSFED OR UFED OR PICA OR rumination disorder  Restrict to: Title |
| Turning Research into Practice Database (TRIP) | (Guideline OR standard OR best practice OR recommendation OR pathway OR policy) AND (Assessment OR recognition OR management OR treatment OR intervention) AND (Perinatal OR pregnan* OR postnatal OR postpartum OR antenatal OR eating disorder OR anorexi* OR bulimi* OR binge eating disorder OR ARFID OR OSFED OR UFED OR PICA OR rumination disorder) |
| Scottish Intercollegiate Guidelines Network (SIGN) | Separate searches for:  Pregnan? .tw.  Perinatal .tw.  Postpartum .tw.  Postnatal .tw.  Eating disorder/ .tw.  Anorexi? .tw.  Bulimi? .tw.  Binge eating disorder/ .tw.  ARFID .tw.  OSFED .tw.  UFED .tw.  PICA .tw.  Rumination disorder/ .tw. |
| International Guidelines Library  World Health Organisation (WHO)  The National Institute for Health and Care Excellence (NICE) | Separate searches for:  ‘pregnan*’  ‘postnatal’  ‘postpartum’  ‘perinatal’  ‘eating disorders’  ‘anorexi*’  ‘bulimi*’  ‘binge eating disorder’  ‘ARFID’  ‘OSFED’  ‘UFED’  ‘PICA’  ‘rumination disorder’ |
| Google | ‘perinatal eating disorder guidelines’  First 100 results |
| Relevant Websites | All websites listed on <https://www.feast-ed.org/worldwide-list-of-ed-advocacy-organizations/> |
