# Supplementary material for: A systematic review of the clinical practice guidelines for the assessment, management and treatment of eating disorders during the perinatal period
Source: BMC Pregnancy Childbirth. 2025 Jan 28;25:82. doi: 10.1186/s12884-024-06995-x (PMC11773850; doi:10.1186/s12884-024-06995-x)
Supplement: Supplementary file 2 — Supplementary Material 2. [file 12884_2024_6995_MOESM2_ESM.docx]

**Additional File 2**

*PICAR Framework for Eligibility Criteria*

| **PICAR Framework** | **Eligibility Criteria** |
| --- | --- |
| **P**opulation, clinical indication(s) and condition(s) | **Study population**   - Women experiencing eating disorders during the perinatal period - CPGs for women in the pre-natal period will be excluded - CPGs for babies of women with EDs postpartum will be excluded   **Clinical indication**   - The assessment, management and treatment of women with eating disorders during the perinatal period   **Clinical condition**   - EDs are defined as psychiatric disorders characterised by abnormal eating or weight-control behaviours (1) - CPGs will be considered if they refer to EDs as a broad category as per the above definition, or if relating to any specific ED diagnosis listed in the DSM-IV (2) or ICD-10 (3) - The perinatal period is defined as the time period between an individual falling pregnant, up until one year after giving birth (32) |
| **I**nterventions | - Any intervention |
| **C**omparator(s), comparison(s) and (key) content | - Any comparator/comparison is of interest - If guidelines are broader in scope, content specific to eating disorders during the perinatal period is only of interest |
| **A**ttributes of the eligible CPGs | **Language**   - CPGs must be available in the English language   **Year of publication**   - No limits on year of publication   **Developing/publishing organisation**   - Must be issued or endorsed by national or international scientific societies, professional colleges, charitable or not-for-profit organisations, or government organisations   **Version**   - Latest version only   **Quality of evidence**   - The eligibility of CPGs were not based on a specific minimum quality cut-off score based on AGREE-II criteria - All CPGs will be considered regardless of quality indicated by the AGREE-II tool   **Scope**   - Must have a focus a primary or secondary focus on assessing, managing or treating eating disorders or women during the perinatal period - The complete/full version of the CPG must be available - Must be clearly labelled as CPGs, guidance or guidelines; position statements and college reports will be excluded |
| **R**ecommendations | - CPGs must report at least one recommendation relating specifically to eating disorders during the perinatal period - CPGs for the assessment, management or treatment of women with eating disorders OR during the perinatal period should be included in full-text screening, to check for specific sections relevant to the research question (e.g. guidelines for eating disorders, where there is a section on the perinatal period OR guidelines for the perinatal period, where there is a section on eating disorders) |
