# Supplementary material for: A systematic review of the clinical practice guidelines for the assessment, management and treatment of eating disorders during the perinatal period
Source: BMC Pregnancy Childbirth. 2025 Jan 28;25:82. doi: 10.1186/s12884-024-06995-x (PMC11773850; doi:10.1186/s12884-024-06995-x)
Supplement: Supplementary file 3 — Supplementary Material 3. [file 12884_2024_6995_MOESM3_ESM.docx]

**Additional File 3**

*Individual AGREE-II item scores per guideline*

|  |  | Domain and Item Numbers | | | | | | | | | | | | | | | | | | | | | | |
| --- | --- | --- | --- | --- | --- | --- | --- | --- | --- | --- | --- | --- | --- | --- | --- | --- | --- | --- | --- | --- | --- | --- | --- | --- |
|  |  | 1. Scope and Purpose | | | 2. Stakeholder Involvement | | | 3. Rigour of Development | | | | | | | | 4. Clarity of Presentation | | | 5. Applicability | | | | 6. Editorial Indepen-dence | |
|  | Guideline Acronym |  |  |  |  |  |  |  |  |  |  |  |  |  |  |  |  |  |  |  |  |  |  |  |
|  |  | 1 | 2 | 3 | 4 | 5 | 6 | 7 | 8 | 9 | 10 | 11 | 12 | 13 | 14 | 15 | 16 | 17 | 18 | 19 | 20 | 21 | 22 | 23 |
| 1 | RCP (2023) | 14 | 14 | 14 | 14 | 14 | 14 | 14 | 14 | 14 | 14 | 14 | 14 | 14 | 14 | 14 | 14 | 14 | 14 | 14 | 14 | 14 | 8 | 14 |
| 2 | UAMS (2023) | 6 | 10 | 10 | 2 | 2 | 10 | 4 | 2 | 2 | 5 | 14 | 2 | 8 | 14 | 7 | 14 | 14 | 2 | 14 | 2 | 2 | 2 | 8 |
| 3 | WFSBP (2023) | 14 | 14 | 10 | 14 | 2 | 2 | 14 | 14 | 14 | 3 | 14 | 14 | 2 | 2 | 7 | 14 | 2 | 4 | 2 | 2 | 2 | 2 | 14 |
| 4 | FNCM (2022) | 14 | 14 | 14 | 14 | 14 | 8 | 12 | 14 | 14 | 14 | 14 | 14 | 14 | 2 | 14 | 14 | 14 | 2 | 2 | 2 | 2 | 14 | 2 |
| 5 | SIGN (2022) | 14 | 14 | 14 | 14 | 14 | 14 | 14 | 14 | 11 | 14 | 14 | 14 | 14 | 14 | 14 | 14 | 14 | 14 | 14 | 8 | 14 | 9 | 14 |
| 6 | BPS (2021) | 14 | 11 | 14 | 7 | 14 | 14 | 2 | 2 | 2 | 3 | 5 | 6 | 14 | 14 | 14 | 14 | 9 | 6 | 2 | 2 | 2 | 2 | 2 |
| 7 | NICE-ED (2020) | 14 | 14 | 14 | 14 | 14 | 14 | 14 | 14 | 14 | 14 | 14 | 2 | 14 | 14 | 14 | 14 | 14 | 14 | 14 | 14 | 14 | 14 | 14 |
| 8 | ANZAED (2020) | 14 | 13 | 14 | 14 | 14 | 14 | 3 | 2 | 2 | 14 | 11 | 14 | 14 | 2 | 14 | 14 | 14 | 14 | 10 | 6 | 5 | 14 | 8 |
| 9 | SADHW (2020) | 14 | 14 | 14 | 4 | 8 | 14 | 4 | 2 | 2 | 2 | 2 | 2 | 2 | 10 | 6 | 10 | 14 | 2 | 2 | 2 | 2 | 2 | 2 |
| 10 | NICE-MH (2020) | 14 | 14 | 14 | 14 | 14 | 14 | 14 | 14 | 8 | 14 | 14 | 2 | 13 | 8 | 14 | 14 | 13 | 14 | 14 | 14 | 14 | 14 | 14 |
| 11 | SOGC (2019) | 14 | 14 | 14 | 14 | 14 | 14 | 14 | 14 | 14 | 14 | 14 | 14 | 2 | 2 | 14 | 14 | 14 | 14 | 14 | 13 | 2 | 2 | 14 |
| 12 | JGG (2018) | 14 | 2 | 12 | 14 | 14 | 6 | 14 | 14 | 14 | 14 | 14 | 14 | 2 | 14 | 14 | 14 | 4 | 14 | 2 | 10 | 2 | 2 | 10 |
| 13 | NEDC (2015) | 6 | 14 | 14 | 10 | 10 | 14 | 2 | 2 | 2 | 2 | 2 | 2 | 8 | 2 | 14 | 14 | 14 | 2 | 12 | 2 | 2 | 2 | 2 |
| 14 | RANZCP (2014) | 14 | 14 | 14 | 6 | 14 | 8 | 14 | 14 | 14 | 14 | 14 | 14 | 14 | 6 | 14 | 14 | 14 | 3 | 4 | 2 | 2 | 14 | 14 |
| 15 | RCOG (2011) | 14 | 14 | 8 | 2 | 2 | 4 | 2 | 2 | 2 | 2 | 14 | 9 | 14 | 7 | 14 | 14 | 14 | 2 | 14 | 2 | 2 | 2 | 4 |
| 16 | FNAH (2010) | 14 | 14 | 14 | 14 | 8 | 14 | 14 | 14 | 14 | 14 | 11 | 2 | 14 | 2 | 14 | 14 | 8 | 3 | 14 | 5 | 8 | 2 | 5 |
| 17 | CAHTA (2009) | 14 | 14 | 14 | 14 | 2 | 14 | 14 | 14 | 11 | 14 | 9 | 2 | 14 | 14 | 14 | 14 | 14 | 5 | 14 | 2 | 14 | 14 | 14 |

*Note.* Scores are the total of the two raters’ scores. Therefore maximum score is 14 and minimum score is 2.
